# A benchmarked protein microarray-based platform for the identification of novel low-affinity extracellular protein interactions

**DOI:** 10.1016/j.ab.2012.01.034

**Published:** 2012-05-01

**Authors:** Yi Sun, Marcus Gallagher-Jones, Colin Barker, Gavin J. Wright

**Affiliations:** Cell Surface Signalling Laboratory, Wellcome Trust Sanger Institute, Hinxton, Cambridge CB10 1HH, UK

**Keywords:** Receptor–ligand pairs, Extracellular protein interactions, AVEXIS, Adhesion receptors, Transient/weak interactions, High-throughput screening, Microarray

## Abstract

Low-affinity extracellular protein interactions are critical for cellular recognition processes, but existing methods to detect them are limited in scale, making genome-wide interaction screens technically challenging. To address this, we report here the miniaturization of the AVEXIS (avidity-based extracellular interaction screen) assay by using protein microarray technology. To achieve this, we have developed protein tags and sample preparation methods that enable the parallel purification of hundreds of recombinant proteins expressed in mammalian cells. We benchmarked the protein microarray-based assay against a set of known quantified receptor–ligand pairs and show that it is sensitive enough to detect even very weak interactions that are typical of this class of interactions. The increase in scale enables interaction screening against a dilution series of immobilized proteins on the microarray enabling the observation of saturation binding behaviors to show interaction specificity and also the estimation of interaction affinities directly from the primary screen. These methodological improvements now permit screening for novel extracellular receptor–ligand interactions on a genome-wide scale.

Extracellular protein–protein interactions between secreted or membrane-tethered proteins are critical for intercellular recognition processes that initiate signaling cascades and provide cellular cohesion within multicellular organisms [1]. These proteins are encoded by approximately 25 to 30% of human genes [2], but despite their abundance and importance, interactions involving these proteins are frequently underrepresented in large-scale systematic protein interaction datasets [3,4]. One plausible reason for this is the unusual biochemical properties of membrane receptor proteins; they are amphipathic, making them difficult to solubilize in their native conformation, and they contain functionally important posttranslational modifications such as disulfide bonds and hydrophilic glycans that are not added in many heterologous and cell-free expression systems. In addition, the interaction affinities between cell surface proteins are typically very low, having half-lives that are less than 1 s [1,5], making them difficult to detect using protocols that involve wash steps. The transient nature of these interactions and the necessity for posttranslational modifications make currently popular high-throughput assays generally unsuitable to detect this class of protein interactions.

Despite the biochemical difficulties of identifying novel low-affinity extracellular receptor–ligand interactions, several scalable methods that take account of some or all of these biochemical challenges have been devised [6–14]. The approach developed by our laboratory, called AVEXIS (avidity-based extracellular interaction screen),^1^ [15] involves expressing the entire extracellular region of membrane-tethered receptors or secreted proteins as soluble recombinant proteins by mammalian cells. The ectodomain fragments are produced as both monobiotinylated baits that can be captured on streptavidin-coated solid phases and β-lactamase-tagged pentamerized preys. The cartilage oligomeric matrix protein (COMP) peptide-mediated pentamerization increases the overall binding avidity to enable reliable detection of interactions having monomeric half-lives of ⩽0.1 s (*K*_D_ ∼ 50 μM) [15], which is within the lower threshold suggested to be of physiological relevance [1,16], whereas β-lactamase enables the enzymatic detection of captured preys. This assay now permits the systematic screening of thousands of binary interactions and has been used to explore cellular recognition events during early vertebrate development [15–19] and host pathogen interactions [20]. However, the current implementation has several disadvantages. First, the preparation of protein libraries using large volumes (>50 ml) of mammalian tissue culture supernatants is labor-intensive and makes storing and sharing these resources difficult. Second, the microtiter plate-based assay uses a comparatively large amount of protein, limiting the size of our interaction screens to just hundreds of proteins. In addition, baits expressed at levels below the assay threshold must be concentrated before use. Finally, excess unconjugated free biotin must be removed by dialysis from the bait preparations before capture on streptavidin-coated microtiter plates. This latter step, in particular, is cumbersome due to the significant number (>100 proteins) and large volumes (∼50 ml) of tissue culture supernatant used.

Protein microarray technology has become a useful tool in the discovery of novel protein–protein interactions, providing a method of capturing thousands of proteins in ordered arrays [21–26]. The use of microarrays has several advantages. Screening is performed in vitro, which importantly provides experimental control over parameters critically affecting assay detection thresholds such as protein concentrations, temperature, washing stringencies, and cofactor dependence. In addition, the miniaturized format requires only tiny amounts of biological sample, and the arrays can be conveniently stored and transported. Protein microarrays have been previously used to detect extracellular protein interactions involved in host–pathogen interactions [27] and low-affinity leukocyte interactions [28]. The proteins that are spotted are usually expressed in bacterial systems (most commonly *Escherichia coli*) or synthesized in situ from arrayed plasmid expression using cell-free expression systems [29–32]. Although convenient, these systems do not add the structurally critical posttranslational modifications such as disulfide bonds or glycans that are necessary to retain the extracellular binding properties of membrane-tethered or secreted proteins.

Here, we show how we have improved our recombinant protein sample preparation workflows and miniaturized the AVEXIS assay so that it can be used on a protein microarray format. Importantly, we benchmarked our protein microarray-based interaction assay against a set of well-characterized low-affinity extracellular protein interactions of known binding strengths and showed that the microarray-based method is at least as sensitive as the plate-based AVEXIS assay but uses approximately five orders of magnitude less protein. This new implementation of the AVEXIS assay will now permit us to increase the size of our interaction screens to a genome-wide scale.

## Materials and methods

### Cell culture and protein expression

**Fig.1:**
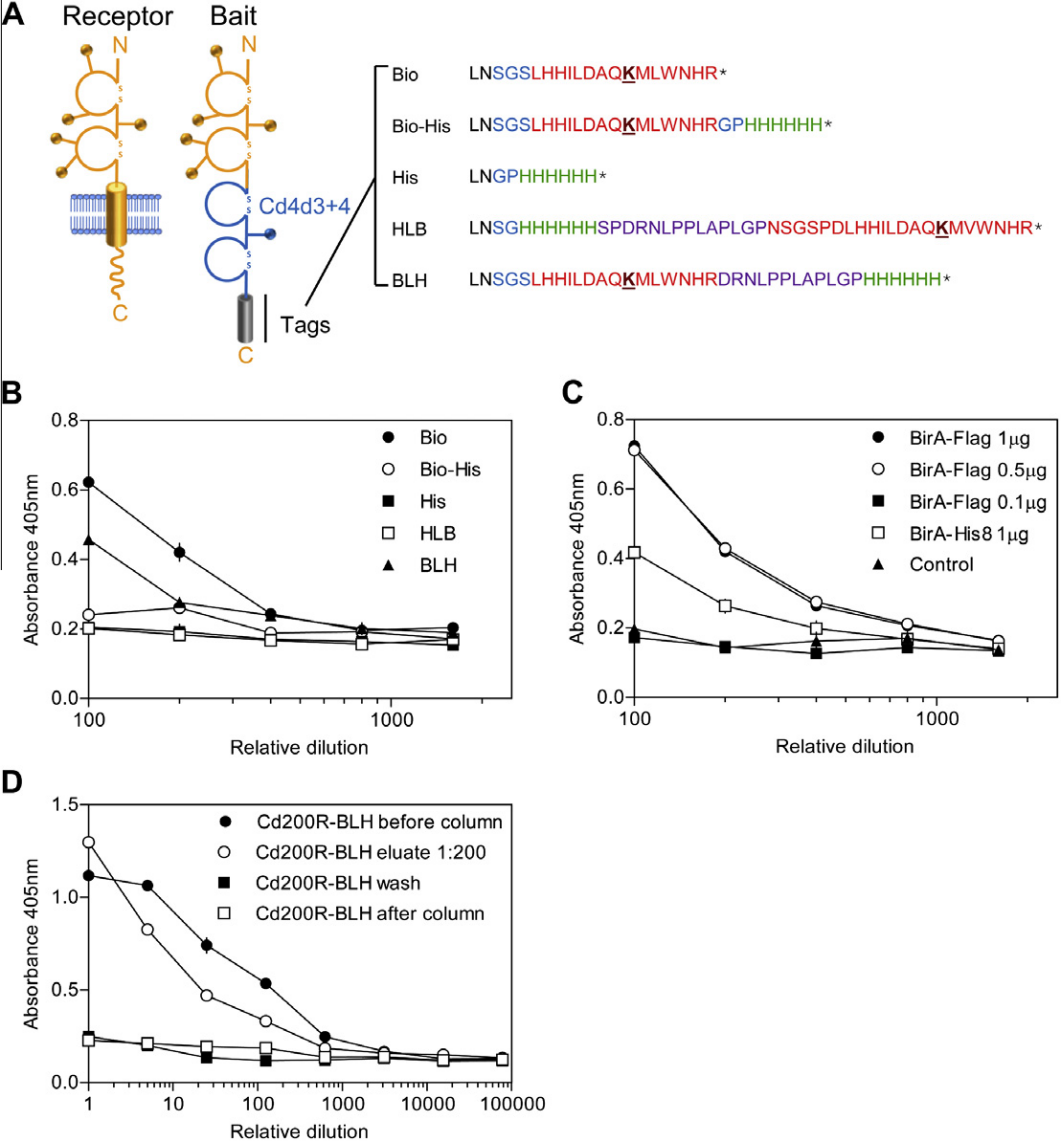
Design of a recombinant protein tag for efficient biotinylation and purification of monomeric proteins for capture on streptavidin-coated surfaces. (A) A schematic diagram showing the sequences of the C-terminal tags used in this study. A cartoon of a typical membrane-embedded type I cell surface receptor is shown next to the corresponding soluble ectodomain expressed as a bait. Lollipops represent potential N-linked glycosylation sites. The sequences of the C-terminal tags are shown and named. “LN” marked in black is the C-terminal end of the Cd4 tag. The peptide sequence that is a substrate for the BirA enzyme is highlighted in red, and the biotinylatable lysine residue is underlined. Flexible linkers and structurally insulating sequences are shown in purple and blue respectively, and oligo-histidine is shown in green. (B) Comparison of the biotinylation efficiencies of a rat Cd200R bait protein containing different C-terminal tags. The different Cd200R bait proteins were expressed with the modified BirA plasmid, normalized to 1 μg/ml, serially diluted on streptavidin-coated microtiter plates, and quantified by ELISA. (C) Cd200R-Bio-l-His bait proteins were cotransfected with a codon-optimized Flag-tagged BirA enzyme (BirA–Flag) or a His8-tagged non-codon-optimized BirA (BirA–His8). Bait expression levels were normalized to 1 μg/ml, and the relative fraction that was biotinylated was determined by ELISA after capture on a streptavidin-coated microtiter plate. (D) Bait proteins containing BLH fusion tag can be efficiently purified on Ni^2+^–NTA resin. Rat Cd200R–BLH baits were expressed and dialyzed to remove free biotin (to enable capture of unpurified supernatants) and then purified using Ni^2+^–NTA resin. Essentially all Cd200R–BLH protein was captured by the Ni^2+^–NTA resin (cf. before and after columns) and was efficiently eluted; little Cd200R–BLH protein leached during washing. Data points in panels B to D represent means ± standard errors (*n *⩾ 3).

All proteins were produced by transient transfection using an HEK293E expression system as described in Refs. [15,33] except that cells were cultured in Freestyle medium (Invitrogen) supplemented with 1% fetal bovine serum. The original bait proteins contained a C-terminal peptide sequence, which in the presence of the *E. coli* enzyme, BirA, and d-biotin is enzymatically monobiotinylated at a specific lysine residue (see “Bio” tag in Fig. 1A). An N-terminal signal peptide was added to the BirA protein to direct it to the secretory pathway, enabling efficient biotinylation of the secreted bait proteins by simply cotransfecting plasmids encoding both the modified BirA enzyme and the bait construct [15,18,19]. Cells were transfected and then incubated for 6 days before supernatants were harvested by removing cells by centrifugation at 3220*g* for 5 min and cell debris by filtering (0.2-μm filter). All samples were stored at 4 °C until use.

### Protein purification

Proteins were purified from spent tissue culture media using Ni^2+^–NTA (nitrilotriacetic acid) resin (GE Healthcare) essentially as described previously [15]. For plate-based purifications, 96-well His MultiTrap HP plates (GE Healthcare) were used in the custom-built, piston-driven sample loading apparatus as described in Additional Data File 1 of the supplementary material. Briefly, plates were spun down to remove any storage solutions and washed once with wash buffer (20 mM phosphate, 0.5 M NaCl, and 40 mM imidazole, pH 7.4) before samples were loaded. After loading was completed, plates were removed and washed twice with wash buffer before being eluted with 100 to 300 μl of elution buffer (20 mM phosphate, 0.5 M NaCl, and 400 mM imidazole, pH 7.4).

### Bait and prey protein normalization

Bait and prey proteins were normalized as described previously [15]. Briefly, concentrations of purified biotinylated bait proteins were determined by enzyme-linked immunosorbent assay (ELISA) on streptavidin-coated plates (Nunc) and then normalized by dilution. The prey proteins were normalized using the β-lactamase enzymatic activity. Prey proteins were normalized by concentrating with 30,000-MWCO (molecular weight cutoff) spin concentrators or diluting in the same manner as the bait proteins. To determine the fraction of protein that was biotinylated, proteins were normalized using an inhibition ELISA with the anti-rat Cd4 monoclonal antibody, OX68 (AbD Serotec).

### Protein microarray production

Normalized bait proteins were diluted in phosphate-buffered saline (PBS) supplemented with 0.02% Tween and 0.5% bovine serum albumin (BSA) prior to printing. Bait proteins were printed on streptavidin-coated slides that also contained an inert hydrogel coating (XanTec) using a MicroGrid II (Digilab) arrayer mounted with 200-μm solid contact pins at 22 °C and 70% relative humidity according to the manufacturer’s instructions. After printing, slides were incubated for 1 h at 70% relative humidity at 22 °C, washed in PBS for 1 h at 22 °C, spin-dried, and stored at −20 °C until use.

### Microarray AVEXIS screening

Prior to interaction screening, printed slides were rinsed three times with MilliQ H_2_O and blocked with PBS containing 1% BSA and 10 mM d-biotin for 45 min. Slides were then incubated with normalized prey proteins for 1 h at 22 °C, washed three times in PBS/0.5% Tween, incubated with 1:1000 anti-Flag horseradish peroxidase (HRP) antibody (Sigma) for 1 h at 22 °C, and finally detected by TSA Alexa 555 substrate (Invitrogen) for 1 h at 22 °C. Slides were washed three times in PBS containing 0.1% Tween 20 with gentle rocking at 22 °C between each incubation step. Positive interactions were identified by scanning slides with a ScanArray Express Microarray Scanner (PerkinElmer) at a 550-nm wavelength.

## Results

### A dual protein tag for recombinant protein purification and capture on streptavidin-coated substrates

Our original AVEXIS assay required producing ectodomain fragments of receptor proteins expressed in mammalian cells as soluble, tagged recombinant biotinylated bait proteins. The need to increase the concentration of bait proteins expressed below the assay threshold and remove excess free biotin from protein preparations, however, prevented expanding the throughput of the assay beyond a few hundred proteins. To address these challenges and increase the scalability of our assay to many hundreds of proteins, we added a 6×His tag at the C terminus of the bait constructs to enable affinity purification on Ni^2+^–NTA resins, thereby removing the need for the dialysis and concentration steps. Initial experiments demonstrated that adding a 6×His tag (Bio-His) directly after the biotin acceptor peptide (Bio) (Fig. 1A) did not support efficient biotinylation compared with the original bait (Fig. 1B). Therefore, we separated the Bio and 6×His tags with a flexible linker sequence (BLH) and made a second version in which the ordering of the peptides was reversed (HLB) (Fig. 1A). To determine the efficiency with which each tag was biotinylated using our experimental system, the ectodomain of the rat Cd200R protein was cloned into vectors containing each of these tags, expressed, and normalized, and the relative fraction of each protein that was biotinylated was quantified by ELISA (Fig. 1B). The BLH tag was more efficiently biotinylated compared with HLB (although not as good as Bio only), suggesting that the local peptide sequence affected the activity of the BirA enzyme for its substrate (Fig. 1B). To further optimize the efficiency of biotinylation and remove the complication that the original secreted *E. coli* BirA enzyme was His8-tagged and, therefore, would be copurified with His-tagged bait proteins [15], we designed a secreted Flag-tagged BirA expression vector that was codon-optimized for mammalian expression. The BirA–Flag construct was more efficient at bait protein biotinylation using our protocol (Fig. 1C). Finally, we showed that the BLH-tagged proteins could be successfully purified using Ni^2+^–NTA resins (Fig. 1D). These refinements in our bait construct design now provide a more convenient system for preparing biotinylated bait protein libraries that can be more easily scaled.

### A cost-effective platform for the convenient parallel purification of bait proteins from large tissue culture volumes

**Fig.2:**
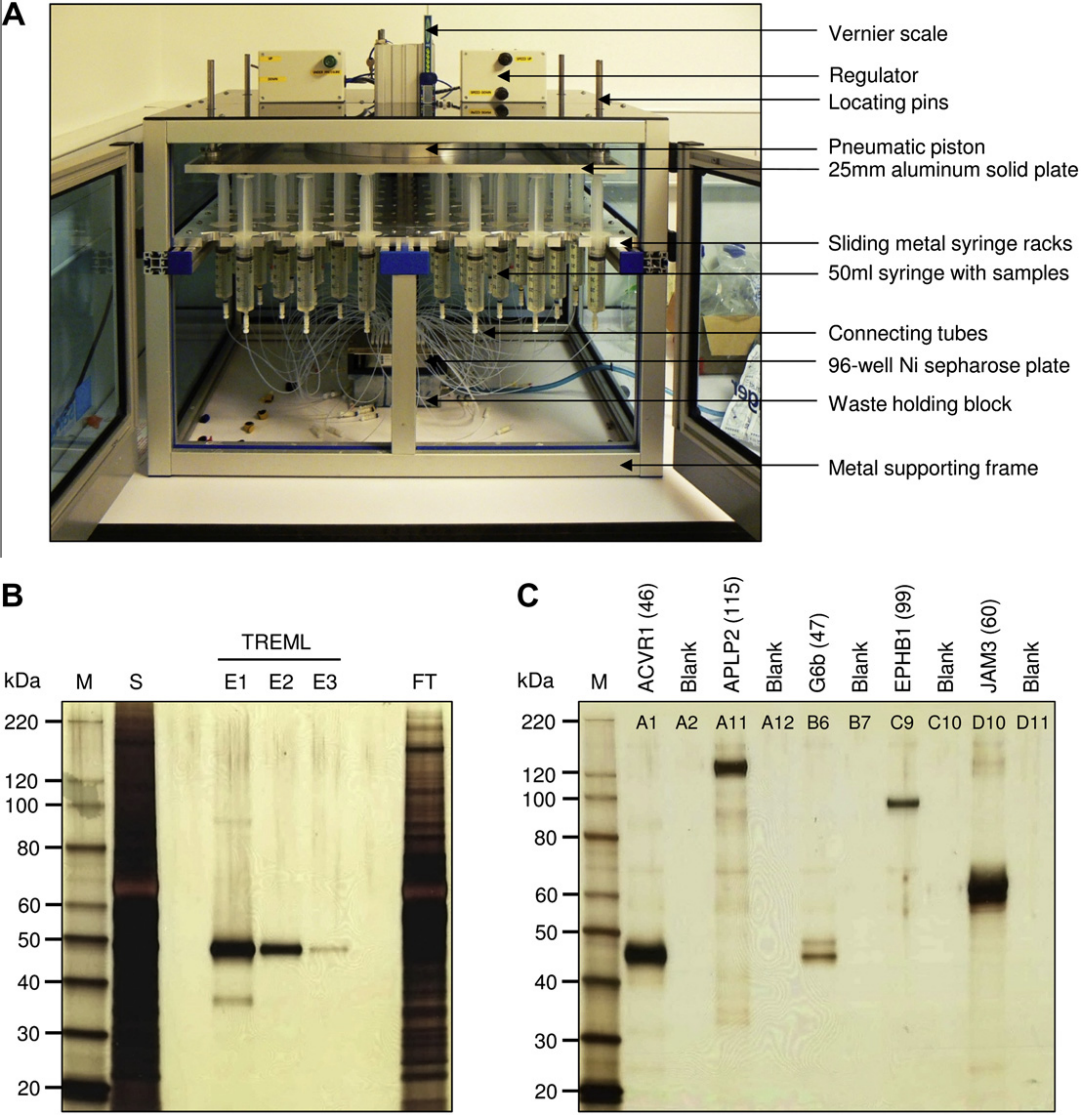
A protein purification system for the parallel purification of 96 large tissue culture volumes. (A) The loading press can purify up to 96 50- to 100-ml samples of His-tagged proteins in parallel. It consists of a pneumatic piston attached to an aluminum plate that is used to drive tissue culture supernatants loaded in 50-ml disposable syringes through tubes that are connected to a holding block containing the 96-well Ni^2+^–NTA resin filter plate. (B) Proteins are purified to greater than 90% purity using the custom loading apparatus. A typical human cell surface receptor protein (TREML) was cloned into the BLH vector and expressed, and 50 ml of spent supernatant was loaded onto a single well of a 96-well microtiter plate containing Ni^2+^–NTA resin. Three serial 200-μl elutions were performed (E1–E3), and 20 μl of a 1:4000 dilution was loaded and resolved by sodium dodecyl sulfate–polyacrylamide gel electrophoresis (SDS–PAGE) under reducing conditions and detected using silver staining. S = 18 μl spent supernatant; FT = 18 μl flow-through; M = markers. (C) No cross-well contamination is detected using the protein purification system. A purification experiment was set up with 48 transfection supernatants and intentionally included blank wells containing tissue culture medium alone to test for cross-well contamination. Eluates from the plate (including neighboring blank wells) were diluted 1:4000, and 20 μl was resolved by SDS–PAGE under reducing conditions and detected by silver staining. The gel shows representative purified bait proteins at the expected mass together with the eluate from neighboring blank wells showing no cross-well contamination.

We have observed, despite a standardized procedure, that individual proteins within our protein libraries are reproducibly expressed in our mammalian expression system at widely varying levels, spanning at least four orders of magnitude [18]. Therefore, low-expressing proteins require larger volumes of tissue culture supernatant (often >50 ml) to produce sufficient amounts for interaction screening. Although His-tagged proteins can be purified in parallel using commercially available Ni^2+^–NTA resins arrayed in 96-well plate formats, they are not designed for sample volumes greater than approximately 500 μl. Therefore, to conveniently purify large numbers of different proteins from 50-ml volumes, we designed and custom-built a purification platform that uses compressed air to power a pneumatic piston delivering supernatants loaded in disposable 50-ml syringes through a 96-well Ni^2+^–NTA plate via microbore tubes at a rate of approximately 1 ml/min (Fig. 2A). This platform enables 96 50- to 100-ml samples to be conveniently purified within a few hours to more than 90% purity (Fig. 2B) without cross-well contamination (Fig. 2C). This cost-effective apparatus enabled the convenient purification of up to 96 transfection supernatants in parallel (full design details can be found in Additional Data File 1).

### Optimization of protein immobilization on streptavidin-coated microarrays

**Fig.3:**
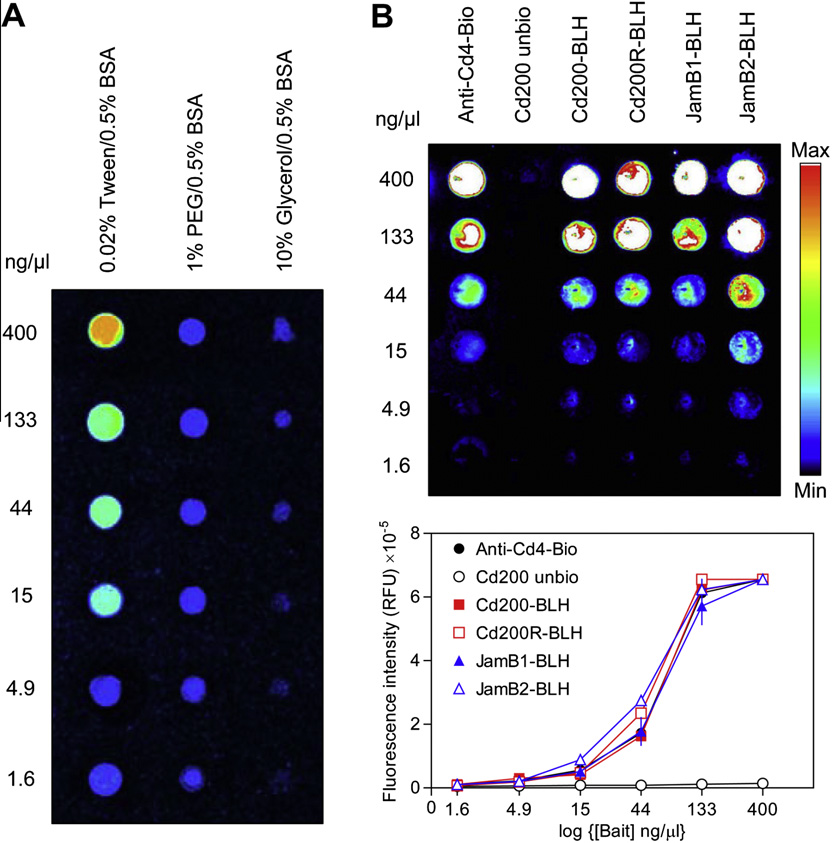
Optimization of biotinylated bait immobilization on streptavidin-coated microarrays. (A) A purified biotinylated Cd200R–BLH protein was serially diluted at the indicated concentrations in three different PBS/0.5% BSA-based buffers before being printed using 400-μm-diameter solid pins on streptavidin-coated slides (XanTec). Buffers were based on a PBS/0.5% BSA solution but contained either 0.02% Tween 20 and 1% polyethylene glycol (PEG, average mass = 8 kDa) or 10% glycerol. The buffer containing 0.02% Tween 20 reproducibly immobilized the most protein with the best spot morphology. (B) The top panel shows a representative image of a protein microarray containing a dilution series of six different proteins that were arrayed on a streptavidin-coated slide before being incubated with an anti-rat Cd4 monoclonal antibody followed by an anti-mouse Cy3-conjugated secondary. The bottom panel graphically shows quantitation of the fluorescence intensities from triplicate arrays on the same slide. Biotinylated bait proteins were specifically captured, as shown by saturation of immobilized bait at 400 μg/ml and lack of immobilization of an unbiotinylated control. The biotinylated anti-Cd4 monoclonal antibody (Anti-Cd4-Bio) serves as a positive control. Data points represent the means ± standard errors (*n *= 3).

To construct protein microarrays containing libraries of bait proteins that were suitable for interaction screening, we first determined the parameters that were important for optimal printing. To ensure that the bait proteins were immobilized in an orientated fashion and in an active conformation, the purified biotinylated bait proteins were equalized, diluted in different buffers, and arrayed on streptavidin-coated microarray slides before being detected using an anti-Cd4 tag monoclonal antibody and a Cy3-conjugated secondary antibody (Fig. 3A). A printing buffer containing 0.02% Tween and 0.5% BSA in PBS reproducibly gave the best results in terms of spot size and morphology using our direct contact solid pin arrayer (Fig. 3A). The amount of bait protein immobilized on the streptavidin-coated slides we used (XanTec) showed evidence of saturation at concentrations of 400 μg/ml, indicating that the proteins were specifically captured via the biotin–streptavidin interaction. A purified but unbiotinylated Cd200 bait protein was not immobilized at 400 μg/ml, demonstrating little capture by passive adsorption (Fig. 3B). Concentrations of ⩾15 μg/ml could be detected on the array with a high-affinity monoclonal antibody recognizing the anti-Cd4 tag.

### Microarray AVEXIS can detect low-affinity interactions

**Fig.4:**
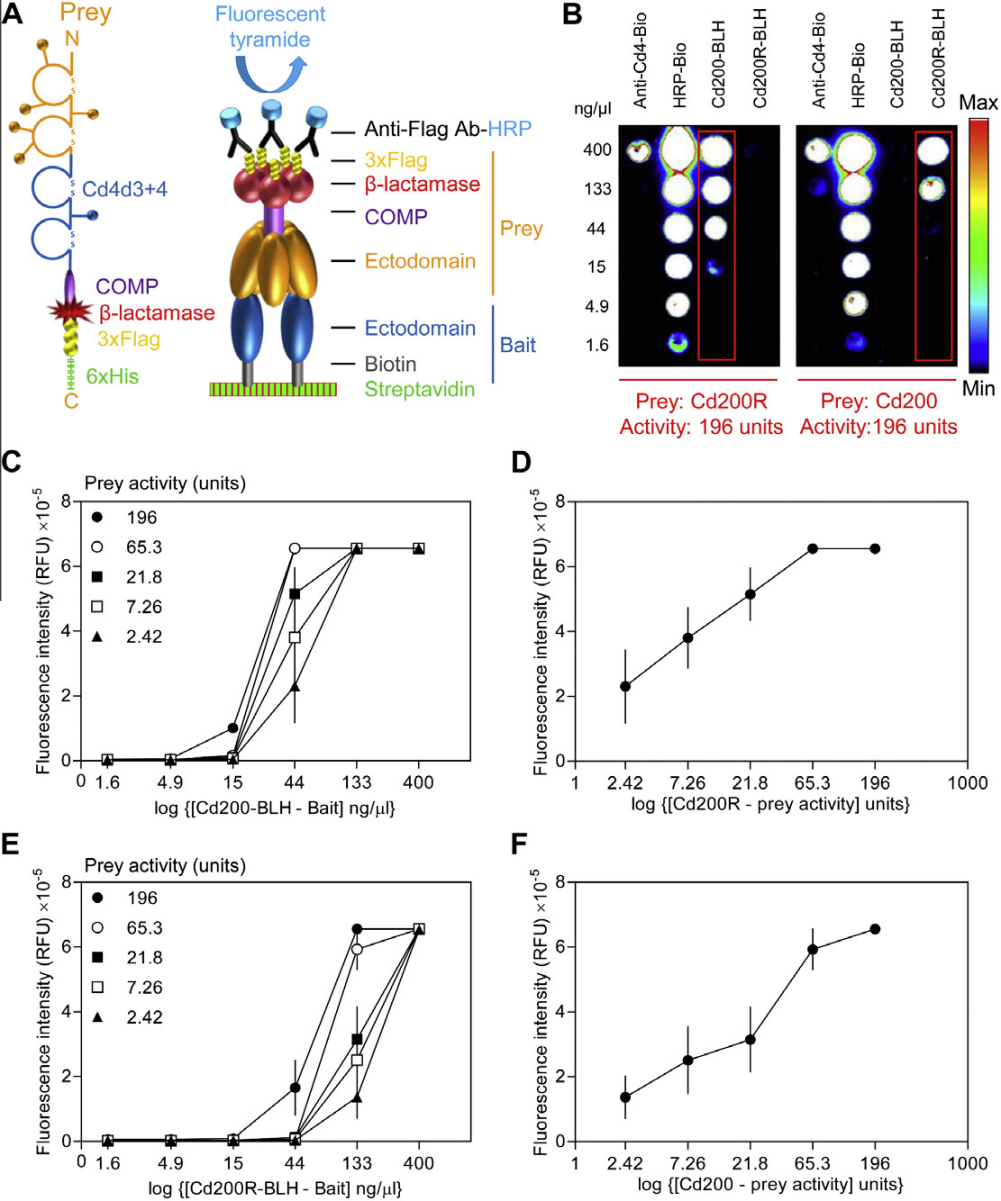
Microarray AVEXIS specifically detects low-affinity extracellular interactions with high sensitivity. (A) A schematic diagram of the pentamerized prey protein and how it is used in the microarray-based AVEXIS assay. Whole ectodomains of cell surface receptor proteins (yellow) are expressed as soluble Cd4-tagged proteins pentamerized by a peptide from the cartilage oligomeric matrix protein (COMP), followed by the β-lactamase enzyme and a triple-Flag tag for detection and a 6×His tag for purification if necessary. The prey is used to probe arrays of bait proteins immobilized on a streptavidin-coated microarray slide. Captured Flag-tagged preys are detected by an anti-Flag HRP-conjugated antibody and then quantified using the deposition of an Alexa Fluor 555 tyramide derivative. (B) Detection of the rat Cd200–Cd200R interaction in both bait–prey orientations by microarray AVEXIS. Serial dilutions of biotinylated rat Cd200–BLH and Cd200R–BLH were immobilized and probed with 196 U of Cd200R (left panel) and CD200 (right panel) preys. The Cd200–Cd200R interaction (within red boxes) was detected in both bait–prey orientations. Serial dilutions of a biotinylated anti-Cd4 antibody (to directly capture the prey via its Cd4 tag) and biotinylated HRP were immobilized as positive controls. (C–F) Determining the sensitivity of the assay to bait and prey activities. Panels C and E show how fluorescence intensity varies according to the bait and prey dilutions when either Cd200 (C) or Cd200R (E) are immobilized as the bait. Panels D and F show a slice through the data shown in panels C and E at bait concentrations of 44 and 133 ng/μl, respectively, illustrating the essentially linear response of the signal to the prey activity. RFU, relative fluorescence units. Data points are means ± standard errors (*n *⩾ 3).

In the original AVEXIS assay, interactions were detected in microtiter plates by probing bait arrays with a pentamerized, β-lactamase-tagged prey protein followed by incubation with a colorimetric soluble β-lactamase substrate, nitrocefin [15]. Because slides are not compartmentalized, detecting interactions on a microarray requires the use of precipitating substrates. Therefore, we modified the prey protein to include a C-terminal 3×Flag–6×His tag that enables detection and, if necessary, purification of the prey protein (Fig. 4A). Captured preys were detected using an anti-Flag HRP-conjugated secondary followed by detection using HRP-activated fluorescent tyramide derivatives that are covalently captured on the microarray. We selected the rat Cd200–Cd200R interaction as a model low-affinity interaction (*K*_D_ = 2 μM, *t*_1/2_ = 0.9 s at 37 °C) [34] to determine the parameters of the assay. A dilution series of both immobilized Cd200 and Cd200R baits were probed with both Cd200 and Cd200R unpurified prey proteins, and the interaction was robustly detected in both bait–prey orientations (Fig. 4B). Importantly, by screening against a dilution series of baits, we observed saturation of the signal at high bait immobilization levels, providing immediate evidence that the detected interaction was specific.

To determine the sensitivity of the assay to the bait and prey concentrations, the bait arrays were probed with a range of prey activities ranging from 196 to 2.42 units, where 1 unit is defined as the amount of prey protein that can turn over 1 nmol of nitrocefin substrate per minute. We observed that the ability to detect interactions was more sensitive to the immobilized bait concentration than the activity of the prey (Figs. 4C–F). For example, diluting a bait that gave a saturated positive detection signal by only 3-fold was sufficient to reduce the signal below the detection threshold (Figs. 4C and 4E). In contrast, signals from a bait dilution that was sensitive to the range of prey activities used were still above the detection threshold despite using an 81-fold dilution range; indeed, the relationship between prey dilution and signal was essentially linear (Figs. 4D and 4F). These results established that bait proteins should be immobilized at concentrations ⩾133 ng/μl to robustly detect a typical low-affinity extracellular protein interaction with an equilibrium dissociation constant within the micromolar (μM) range. We also used this experiment to select a minimum prey activity of 65.3 U for our subsequent screens.

### Quantitative benchmarking of the microarray AVEXIS using interactions with the zebrafish Jam family

**Fig.5:**
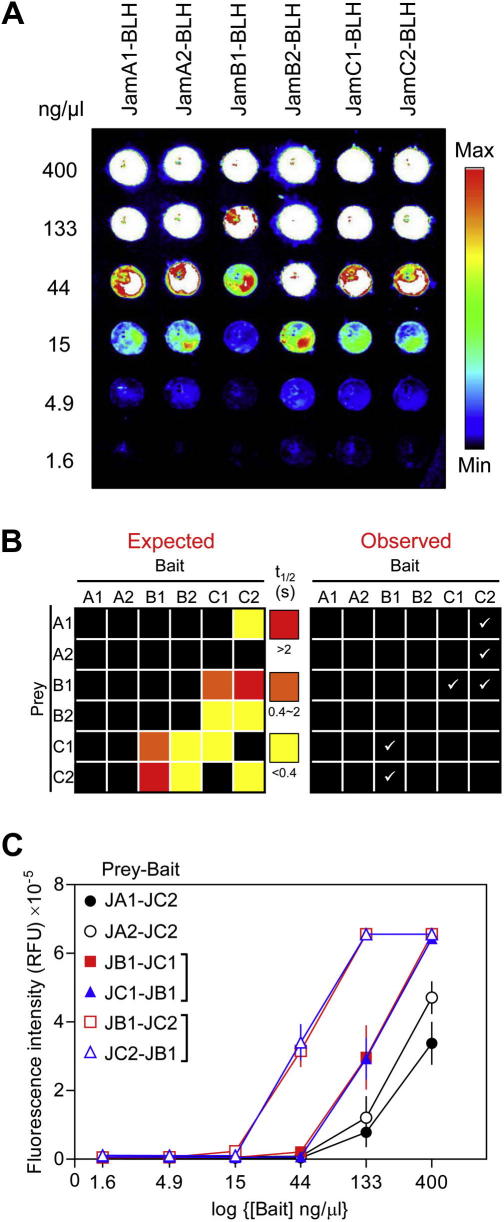
Quantitative benchmarking of the microarray AVEXIS using interactions within the zebrafish Jam family. (A) All six members of the zebrafish Jam family were expressed as biotinylated baits, purified, normalized, serially diluted, and arrayed on streptavidin-coated slides before detection with an anti-Cd4 primary antibody and a Cy3-conjugated goat anti-mouse secondary antibody. The image shows that similar levels of Jam bait proteins were immobilized on the slide. (B) Graphical representation of expected and observed interactions of different affinities within the zebrafish Jam family. The expected (left panel) interactions within the zebrafish Jam family were grouped according to their affinities (red = high affinity [*t*_1/2_ > 2 s]; orange = medium affinity [0.4 < *t*_1/2_ < 2 s]; yellow = low affinity [*t*_1/2_ < 0.4 s]). The observed interactions using the microarray screening approach are depicted graphically in the right panel. (C) The affinity of interactions detected on the microarray correlated with the sensitivity to bait immobilization level in both bait–prey orientations. The ability to detect the four interactions within the Jam family as a function of the immobilized bait level was determined using microarray AVEXIS. Higher affinity interactions (e.g., JamB1–JamC2) were less sensitive to the bait immobilization level, and the same interaction showed the same sensitivity to the bait immobilization level in both bait–prey orientations. RFU, relative fluorescence units. Data points are means ± standard errors (*n *⩾ 3).

Although the rat Cd200–Cd200R interaction has a relatively low affinity with a K_D_ of approximately 2 μM, there are several reported receptor–ligand interactions that are considerably weaker than this [35,36] and it has been estimated that interactions as weak as 50 μM (*t*_1/2_ ∼ 0.1 s) are sufficient to drive spontaneous alignment of apposing membranes [16]. To quantify the sensitivity limit of our assay, we screened for interactions within the family of six paralogous receptor proteins belonging to the zebrafish junctional cell adhesion molecule (JAM) family (Fig. 5A). Results from our group have shown, using mutant zebrafish and cellular transplantation studies, that the interaction between JamB1 and JamC1 is necessary for myoblast fusion in vivo [37]. We have also systematically quantified the monomeric interaction strengths among all six proteins using surface plasmon resonance (SPR) (Powell et al., in preparation), providing us with a quantified set of interactions whose strengths range from the relatively high-affinity JamB1–JamC2 interaction (*t*_1/2_ > 2 s) to the JamA1–JamC2 interaction that can be barely detected (*t*_1/2_ < 0.4 s) (Fig. 5B). All six Jam family bait proteins were immobilized as a dilution series (Fig. 5A) and probed using a set of the corresponding six pentameric preys. The JamB1–JamC1 interaction (*t*_1/2_ = 1.9 s), which is known to be physiologically relevant in vivo [37], was robustly detected (Fig. 5B), as was the higher affinity JamB1–JamC2 interaction (*t*_1/2_ > 2 s). Both of these expected interactions were detected in both bait–prey orientations. The very weak JamC2–JamA1 interaction (*t*_1/2_ < 0.4 s) was detected in just one bait–prey orientation, and an interaction between JamA2 and JamC2 that had not been detected by our SPR studies was now observed, again in just one orientation (Fig. 5B). The very weak (*t*_1/2_ < 0.4 s) JamB2–JamC1 and JamB2–JamC2 interactions were not detected. None of the homophilic interactions was detected, possibly due to highly avid prey–prey interactions that prevent prey–bait interactions; this prey “masking” effect was also observed in the plate-based assay [15]. Interestingly, the strength of the interaction correlated with the ability to detect the interaction at lower bait concentrations; for example, the strong JamB1–JamC2 interaction was easily detected at a bait concentration of 44 ng/μl, whereas the weaker interactions were not (Fig. 5C). This suggests that it may be possible to estimate an approximate affinity for an interaction during the primary screen by probing a serial dilution of bait proteins despite the unpredictable nature of avidity gains achieved by multimerization. We also observed that the JamB1–JamC2 and JamB1–JamC1 interaction exhibited the same sensitivity to the level of bait immobilization regardless of the bait–prey orientation used to detect it (Fig. 5C). This suggests that the bait and prey activities (fraction of functional protein) are comparable for each interaction. Although this might be expected for the prey protein in solution, others have observed an unexpectedly wide range of protein activities when similar proteins are immobilized on a solid substrate [38].

## Discussion

Defining protein interaction networks on an increasingly large scale to understand how biological processes are coordinately regulated has been a focus of recent research interest. These efforts have relied on a small number of high-throughput assays such as biochemical purification followed by mass spectrometry [39,40], yeast two-hybrid screening [41,42], and other protein complementation assays [43]. Although these approaches have revealed a great deal regarding the architecture of intracellular protein interaction networks, none of these assays is generally suitable for detecting low-affinity extracellular interactions. To address this problem, several scalable assays specifically designed to detect extracellular interactions have been developed [6,9,11–13,15] but are practically limited to screening modest library sizes of several hundred receptor proteins, whereas vertebrate genomes are known to contain several thousand [1]. Here, we have shown that it is possible to implement the AVEXIS assay as a protein microarray format, which, together with improved sample handling and the development of custom-built protein sample preparation hardware, makes extracellular protein interaction screening feasible on a genome-wide scale.

Miniaturizing the AVEXIS assay on a microarray compared with the microtiter plate format has several advantages. The amount of protein required per interaction test has been dramatically reduced by approximately five orders of magnitude. This now reduces the amount of sample preparation time required and is particularly important for those proteins that are expressed at low levels. It also enables an increase in the number of interaction screens that can be performed with the same protein preparation, enabling more replicates to be performed with a wider range of protein concentrations. This latter point is of particular value because by screening against a dilution series of bait proteins it was possible to determine whether binding events were saturable (and therefore specific) and to estimate a binding affinity directly from the screen. The observation that the known binding affinity of an interaction correlated with its dose sensitivity across the bait dilutions used was a surprise given the unpredictable gain in binding avidity caused by multimerization. This is likely due to the fact that we used a similar structural class of receptor proteins in the experiments described here (all are type I cell surface proteins containing two immunoglobulin superfamily domains that interact with a 1:1 stoichiometry [34,37]), and we would not expect this to be generally true of receptors belonging to different structural classes. An important aspect of the approach described here is the quantitative determination of the assay detection thresholds using variable parameters such as the bait and prey levels. By using a panel of known interaction strengths, we determined that our microarray-based assay is capable of detecting interactions that are known to be physiologically relevant and that are thought to be on the lower limit for in vivo significance. Finally, a microarray is more convenient for storing and distributing a large number of proteins in a format that is suitable for a range of different screening approaches.

The development of the dual biotin–His tag and the custom loading apparatus has significantly improved the throughput and convenience of protein sample preparation when using the large supernatant volumes required by mammalian expression systems. The purification of the biotinylated proteins using the oligo–His tag has removed the need for the cumbersome dialysis steps and has the additional advantage that the proteins can be eluted from the microtiter plate in small (50–300 μl) volumes so that very high concentrations (>10 mg/ml) of purified proteins can be achieved. This approach also retains the advantage of irreversibly capturing the monobiotinylated bait proteins on streptavidin-coated solid phases in an orientated manner that, as we showed here, may reduce the wide variance in immobilized protein activity that has been observed when proteins are captured nonspecifically by passive adsorption [38]. Retaining the β-lactamase enzyme in the prey construct enables rapid quantification of the prey activity to facilitate prey normalization prior to addition to the slides and is fully compatible with performing the assay on microtiter plates. The rat Cd4d3 + 4 tag in both the bait and prey provides flexibility in using other antibody-based methods for normalizing the protein expression levels, and although the presence of the Cd4d3 + 4 tag in the prey might seem redundant, we found it to be useful when used together with a biotinylated anti-Cd4 monoclonal antibody as a prey capture positive control (as shown in Figs. 3 and 4). The Cd4d3 + 4 tag [44] has been used by several laboratories over a number of years without any reports of unexpected interactions [34,45–49]. The addition of a His-tag C terminal to the β-lactamase also enables purification of the prey in a similar manner to the baits. Although we showed that it is not necessary to purify the prey proteins for screening, this now provides the ability to easily concentrate poorly expressing prey proteins by factors greater than 500-fold to levels that are sufficient to be included in screens. Previously, low-expressing prey protein solutions were concentrated using centrifugation filters that limited concentration factors to approximately 10-fold. The loading device described in our article can be constructed on a limited budget (∼$5000), making it accessible to a wide range of laboratories. It is compatible with any standard 96-well plate format and, therefore, can be used for a range of applications such as purifying antibodies on protein G or protein A plates.

The arrayed receptor protein microarray slides are not limited to the study of extracellular protein interactions. We envisage that these receptor arrays will also be very useful for systematically studying interactions using whole cells or pathogens such as bacteria and viruses. In addition, these slides could be used to screen complex biological tissues such as serum samples for the identification of diagnostic disease markers.

In conclusion, by developing new protein tags and streamlining the sample preparation procedures, we have used protein microarrays to miniaturize and improve the AVEXIS assay that is capable of detecting low-affinity extracellular protein interactions. By reducing the amount of protein required, the size of the screens that can be performed has increased significantly. We determined the experimental parameters necessary to detect interactions as weak as 50 μM, which are considered as a lower limit for physiological significance [1,16]. The microarray has advantages over the plate-based format of estimating interaction affinities and observing saturable (specific) binding behaviors during the initial screen, thereby providing immediate additional criteria for selecting which interactions should be prioritized for further study. The human genome is believed to contain approximately 2000 receptor and secreted proteins that would be suitable for interaction screening using the AVEXIS approach and whose extracellular interactions are unlikely to be detected using other scalable protein interaction techniques [1]. Because the extracellular regions of receptor proteins are accessible to systematically delivered drugs such as therapeutic antibodies, they are often described as “druggable” targets, making systematically determined extracellular protein interaction networks of particular importance. The miniaturization of the AVEXIS assay and its technological improvements now make whole-genome screening for low-affinity extracellular protein interactions a possibility.

## Footnotes

1 *Abbreviations used:* AVEXIS, avidity-based extracellular interaction screen; NTA, nitrilotriacetic acid; ELISA, enzyme-linked immunosorbent assay; PBS, phosphate-buffered saline; BSA, bovine serum albumin; HRP, horseradish peroxidase; SPR, surface plasmon resonance.

## References

[1] Wright G.J. (2009). Signal initiation in biological systems: The properties and detection of transient extracellular protein interactions. Mol. Biosyst..

[2] Fagerberg L., Jonasson K., von Heijne G., Uhlen M., Berglund L. (2010). Prediction of the human membrane proteome. Proteomics.

[3] Braun P., Tasan M., Dreze M., Barrios-Rodiles M., Lemmens I., Yu H., Sahalie J.M., Murray R.R., Roncari L., de Smet A.S., Venkatesan K., Rual J.F., Vandenhaute J., Cusick M.E., Pawson T., Hill D.E., Tavernier J., Wrana J.L., Roth F.P., Vidal M. (2009). An experimentally derived confidence score for binary protein–protein interactions. Nat. Methods.

[4] Futschik M.E., Chaurasia G., Herzel H. (2007). Comparison of human protein–protein interaction maps. Bioinformatics.

[5] van der Merwe P.A., Barclay A.N. (1994). Transient intercellular adhesion: The importance of weak protein–protein interactions. Trends Biochem. Sci..

[6] de Wildt R.M., Tomlinson I.M., Ong J.L., Holliger P. (2002). Isolation of receptor–ligand pairs by capture of long-lived multivalent interaction complexes. Proc. Natl. Acad. Sci. USA.

[7] Faye C., Chautard E., Olsen B.R., Ricard-Blum S. (2009). The first draft of the endostatin interaction network. J. Biol. Chem..

[8] Gonzalez L.C., Loyet K.M., Calemine-Fenaux J., Chauhan V., Wranik B., Ouyang W., Eaton D.L. (2005). A coreceptor interaction between the CD28 and TNF receptor family members B and T lymphocyte attenuator and herpesvirus entry mediator. Proc. Natl. Acad. Sci. USA.

[9] Jiang L., Barclay A.N. (2010). Identification of leucocyte surface protein interactions by high-throughput screening with multivalent reagents. Immunology.

[10] Jiang L., Barclay A.N. (2009). New assay to detect low-affinity interactions and characterization of leukocyte receptors for collagen including leukocyte-associated Ig-like receptor-1 (LAIR-1). Eur. J. Immunol..

[11] Urech D.M., Lichtlen P., Barberis A. (2003). Cell growth selection system to detect extracellular and transmembrane protein interactions. Biochim. Biophys. Acta.

[12] Voulgaraki D., Mitnacht-Kraus R., Letarte M., Foster-Cuevas M., Brown M.H., Barclay A.N. (2005). Multivalent recombinant proteins for probing functions of leucocyte surface proteins such as the CD200 receptor. Immunology.

[13] Wojtowicz W.M., Wu W., Andre I., Qian B., Baker D., Zipursky S.L. (2007). A vast repertoire of Dscam binding specificities arises from modular interactions of variable Ig domains. Cell.

[14] J.D. Humphries, A. Byron, M.D. Bass, S.E. Craig, J.W. Pinney, D. Knight, M.J. Humphries, Proteomic analysis of integrin-associated complexes identifies RCC2 as a dual regulator of Rac1 and Arf6, Sci. Signal. 2 (2009) ra51. 10.1126/scisignal.2000396 PMC2857963 19738201

[15] Bushell K.M., Sollner C., Schuster-Boeckler B., Bateman A., Wright G.J. (2008). Largescale screening for novel low-affinity extracellular protein interactions. Genome Res..

[16] Dustin M.L., Golan D.E., Zhu D.M., Miller J.M., Meier W., Davies E.A., van der Merwe P.A. (1997). Low affinity interaction of human or rat T cell adhesion molecule CD2 with its ligand aligns adhering membranes to achieve high physiological affinity. J. Biol. Chem..

[17] Charoensawan V., Adryan B., Martin S., Sollner C., Thisse B., Thisse C., Wright G.J., Teichmann S.A. (2010). The impact of gene expression regulation on evolution of extracellular signaling pathways. Mol. Cell. Proteomics.

[18] Martin S., Sollner C., Charoensawan V., Adryan B., Thisse B., Thisse C., Teichmann S., Wright G.J. (2010). Construction of a large extracellular protein interaction network and its resolution by spatiotemporal expression profiling. Mol. Cell. Proteomics.

[19] Sollner C., Wright G.J. (2009). A cell surface interaction network of neural leucine-rich repeat receptors. Genome Biol..

[20] Crosnier C., Bustamante L.Y., Bartholdson S.J., Bei A.K., Theron M., Uchikawa M., Mboup S., Ndir O., Kwiatkowski D.P., Duraisingh M.T., Rayner J.C., Wright G.J. (2011). Basigin is a receptor essential for erythrocyte invasion by *Plasmodium falciparum*. Nature.

[21] Cahill D.J., Nordhoff E. (2003). Protein arrays and their role in proteomics. Adv. Biochem. Eng. Biotechnol..

[22] Fasolo J., Sboner A., Sun M.G., Yu H., Chen R., Sharon D., Kim P.M., Gerstein M., Snyder M. (2011). Diverse protein kinase interactions identified by protein microarrays reveal novel connections between cellular processes. Genes Dev..

[23] Merbl Y., Kirschner M.W. (2009). Large-scale detection of ubiquitination substrates using cell extracts and protein microarrays. Proc. Natl. Acad. Sci. USA.

[24] Popescu S.C., Snyder M., Dinesh-Kumar S. (2007). *Arabidopsis* protein microarrays for the high-throughput identification of protein–protein interactions. Plant Signal. Behav..

[25] Stoevesandt O., Taussig M.J., He M. (2009). Protein microarrays: High-throughput tools for proteomics. Expert Rev. Proteomics.

[26] Wolf-Yadlin A., Sevecka M., MacBeath G. (2009). Dissecting protein function and signaling using protein microarrays. Curr. Opin. Chem. Biol..

[27] Margarit I., Bonacci S., Pietrocola G., Rindi S., Ghezzo C., Bombaci M., Nardi-Dei V., Grifantini R., Speziale P., Grandi G. (2009). Capturing host–pathogen interactions by protein microarrays: Identification of novel streptococcal proteins binding to human fibronectin, fibrinogen, and C4BP. FASEB J..

[28] Letarte M., Voulgaraki D., Hatherley D., Foster-Cuevas M., Saunders N.J., Barclay A.N. (2005). Analysis of leukocyte membrane protein interactions using protein microarrays. BMC Biochem..

[29] He M., Stoevesandt O., Palmer E.A., Khan F., Ericsson O., Taussig M.J. (2008). Printing protein arrays from DNA arrays. Nat. Methods.

[30] He M., Taussig M.J. (2001). Single step generation of protein arrays from DNA by cell-free expression and in situ immobilisation (PISA method). Nucleic Acids Res..

[31] Ramachandran N., Hainsworth E., Bhullar B., Eisenstein S., Rosen B., Lau A.Y., Walter J.C., LaBaer J. (2004). Self-assembling protein microarrays. Science.

[32] Ramachandran N., Raphael J.V., Hainsworth E., Demirkan G., Fuentes M.G., Rolfs A., Hu Y., LaBaer J. (2008). Next-generation high-density self-assembling functional protein arrays. Nat. Methods.

[33] Durocher Y., Perret S., Kamen A. (2002). High-level and high-throughput recombinant protein production by transient transfection of suspension-growing human 293-EBNA1 cells. Nucleic Acids Res..

[34] Wright G.J., Puklavec M.J., Willis A.C., Hoek R.M., Sedgwick J.D., Brown M.H., Barclay A.N. (2000). Lymphoid/neuronal cell surface OX2 glycoprotein recognizes a novel receptor on macrophages implicated in the control of their function. Immunity.

[35] Mavaddat N., Mason D.W., Atkinson P.D., Evans E.J., Gilbert R.J., Stuart D.I., Fennelly J.A., Barclay A.N., Davis S.J., Brown M.H. (2000). Signaling lymphocytic activation molecule (CDw150) is homophilic but self-associates with very low affinity. J. Biol. Chem..

[36] van der Merwe P.A., Brown M.H., Davis S.J., Barclay A.N. (1993). Affinity and kinetic analysis of the interaction of the cell adhesion molecules rat CD2 and CD48. EMBO J..

[37] Powell G.T., Wright G.J. (2011). JamB and JamC are essential for vertebrate myocyte fusion. PLoS Biol..

[38] Gordus A., MacBeath G. (2006). Circumventing the problems caused by protein diversity in microarrays: Implications for protein interaction networks. J. Am. Chem. Soc..

[39] Gavin A.C., Bosche M., Krause R., Grandi P., Marzioch M., Bauer A., Schultz J., Rick J.M., Michon A.M., Cruciat C.M., Remor M., Hofert C., Schelder M., Brajenovic M., Ruffner H., Merino A., Klein K., Hudak M., Dickson D., Rudi T., Gnau V., Bauch A., Bastuck S., Huhse B., Leutwein C., Heurtier M.A., Copley R.R., Edelmann A., Querfurth E., Rybin V., Drewes G., Raida M., Bouwmeester T., Bork P., Seraphin B., Kuster B., Neubauer G., Superti-Furga G. (2002). Functional organization of the yeast proteome by systematic analysis of protein complexes. Nature.

[40] Krogan N.J., Cagney G., Yu H., Zhong G., Guo X., Ignatchenko A., Li J., Pu S., Datta N., Tikuisis A.P., Punna T., Peregrin-Alvarez J.M., Shales M., Zhang X., Davey M., Robinson M.D., Paccanaro A., Bray J.E., Sheung A., Beattie B., Richards D.P., Canadien V., Lalev A., Mena F., Wong P., Starostine A., Canete M.M., Vlasblom J., Wu S., Orsi C., Collins S.R., Chandran S., Haw R., Rilstone J.J., Gandi K., Thompson N.J., Musso G., St. Onge P., Ghanny S., Lam M.H., Butland G., Altaf-Ul A.M., Kanaya S., Shilatifard A., O’Shea E., Weissman J.S., Ingles C.J., Hughes T.R., Parkinson J., Gerstein M., Wodak S.J., Emili A., Greenblatt J.F. (2006). Global landscape of protein complexes in the yeast *Saccharomyces cerevisiae*. Nature.

[41] Rual J.F., Venkatesan K., Hao T., Hirozane-Kishikawa T., Dricot A., Li N., Berriz G.F., Gibbons F.D., Dreze M., Ayivi-Guedehoussou N., Klitgord N., Simon C., Boxem M., Milstein S., Rosenberg J., Goldberg D.S., Zhang L.V., Wong S.L., Franklin G., Li S., Albala J.S., Lim J., Fraughton C., Llamosas E., Cevik S., Bex C., Lamesch P., Sikorski R.S., Vandenhaute J., Zoghbi H.Y., Smolyar A., Bosak S., Sequerra R., Doucette-Stamm L., Cusick M.E., Hill D.E., Roth F.P., Vidal M. (2005). Towards a proteome-scale map of the human protein–protein interaction network. Nature.

[42] Stelzl U., Worm U., Lalowski M., Haenig C., Brembeck F.H., Goehler H., Stroedicke M., Zenkner M., Schoenherr A., Koeppen S., Timm J., Mintzlaff S., Abraham C., Bock N., Kietzmann S., Goedde A., Toksoz E., Droege A., Krobitsch S., Korn B., Birchmeier W., Lehrach H., Wanker E.E. (2005). A human protein–protein interaction network: A resource for annotating the proteome. Cell.

[43] Tarassov K., Messier V., Landry C.R., Radinovic S., Serna Molina M.M., Shames I., Malitskaya Y., Vogel J., Bussey H., Michnick S.W. (2008). An in vivo map of the yeast protein interactome. Science.

[44] Brown M.H., Barclay A.N. (1994). Expression of immunoglobulin and scavenger receptor superfamily domains as chimeric proteins with domains 3 and 4 of CD4 for ligand analysis. Protein Eng..

[45] Hargreaves P.G., Al-Shamkhani A. (2002). Soluble CD30 binds to CD153 with high affinity and blocks transmembrane signaling by CD30. Eur. J. Immunol..

[46] Mason D.Y., Cordell J.L., Brown M.H., Borst J., Jones M., Pulford K., Jaffe E., Ralfkiaer E., Dallenbach F., Stein H., Pileri S., Gatter K.C. (1995). CD79a: A novel marker for B-cell neoplasms in routinely processed tissue samples. Blood.

[47] Schofield D.J., Pope A.R., Clementel V., Buckell J., Chapple S., Clarke K.F., Conquer J.S., Crofts A.M., Crowther S.R., Dyson M.R., Flack G., Griffin G.J., Hooks Y., Howat W.J., Kolb-Kokocinski A., Kunze S., Martin C.D., Maslen G.L., Mitchell J.N., O’Sullivan M., Perera R.L., Roake W., Shadbolt S.P., Vincent K.J., Warford A., Wilson W.E., Xie J., Young J.L., McCafferty J. (2007). Application of phage display to high throughput antibody generation and characterization. Genome Biol..

[48] Tan S.M., Walters S.E., Mathew E.C., Robinson M.K., Drbal K., Shaw J.M., Law S.K. (2001). Defining the repeating elements in the cysteine-rich region (CRR) of the CD18 integrin β2 subunit. FEBS Lett..

[49] Wright G.J., Giudicelli F., Soza-Ried C., Hanisch A., Ariza-McNaughton L., Lewis J. (2011). DeltaC and DeltaD interact as Notch ligands in the zebrafish segmentation clock. Development.

