# Supplementary material for: A benchmarked protein microarray-based platform for the identification of novel low-affinity extracellular protein interactions
Source: Anal Biochem. 2012 May 1;424(1-3):45–53. doi: 10.1016/j.ab.2012.01.034 (PMC3325482; doi:10.1016/j.ab.2012.01.034)
Supplement: Supplementary data 1 — Supplementary material [file mmc1.pdf]

Additional data file 1 for:

A benchmarked protein microarray-based platform for the identification of novel low affinity extracellular protein interactions.

Yi Sun, Marcus Gallagher-Jones, Colin Barker and Gavin J. Wright

Design and construction of a bespoke loading apparatus.

All recombinant proteins for our binding studies are produced as secreted recombinant proteins using a mammalian expression system. Proteins are expressed by transient transfection resulting in the expression of 6His-tagged proteins in spent tissue culture in a volume of 50 ml. To purify a large number of His-tagged proteins in parallel, we used a commercially available 96-well microtitre plate containing Ni^{2+}-NTA resin (His MultiTrap 96-well filter plate, GE Healthcare). However, because the holding volume of each well is limited to only ~ 500 microlitres, this makes loading 50 ml of supernatant through each well challenging. To address this, we designed and built a bespoke loading press.

The press was designed to be operated on a normal laboratory bench using pneumatics and consists of a frame constructed of 40x40 aluminium speed frame (Rose & Krieger), a pneumatic piston (we used a C(D)Q2, Compact Cylinder, Double Acting, Single Rod (SMC Pneumatics, UK)) attached to an ~ 25 mm thick aluminium plate and a holding base to contain the 96-well filter plate (Fig. S1A). The pneumatics system is required to have sufficient force to compress 96 fully-loaded 50ml syringes at a controlled speed of approx 1 ml min^{-1}, which is the standard flow rate for protein purification using this resin. To achieve the required speed, high grade precision needle valves (SMC Pneumatics, UK) were used as the regulators. The movement of the pressure plate is relatively slow when in operation, so a set of digital vernier calipers were attached between the pressure plate and the main frame to enable visual indication and quantification of the movement rate. The transfection supernatants were loaded into 50 ml disposable plastic syringes and sited into metal loading bars (Fig. S1B, S1C) that were removable and therefore allowed sequential and convenient loading of all 96 syringes into the frame.

The filter plate assembly and fittings are made of aluminium and consist of a lower block with a drain outlet and a top plate held in position using four locator pins (Fig. S1D, S1E). The Ni^{2+}-NTA filter plate is fixed between these two and bounded by two silicon membranes to ensure a tight seal and prevent well-to-well leakage (Fig. S1F). The top plate was drilled to make 96

tapped holes at a spacing that corresponded to the wells in the filter plate (Fig. S1G, S1H, S1I). Tubing and fittings were purchased from PTFE Parts Ltd, Cambridgeshire, UK: 1.6mm outer diameter, 1.0 mm microbore PTFE tubing were fixed to the top plate with acetal fittings and cone seals (Cat. Nos. 4-SPAN-001 and 4-PP-1012B). The other end of the tubing had a luer connection made using a microbore tubing adapter, end connector and fitting (Cat. Nos. 2-PP-0012-TUB, 4-PP-1056, 4-VP-BSFTLL-6) that could be attached directly to the syringes. Each connection was colour-coded and numbered to facilitate correct positioning.

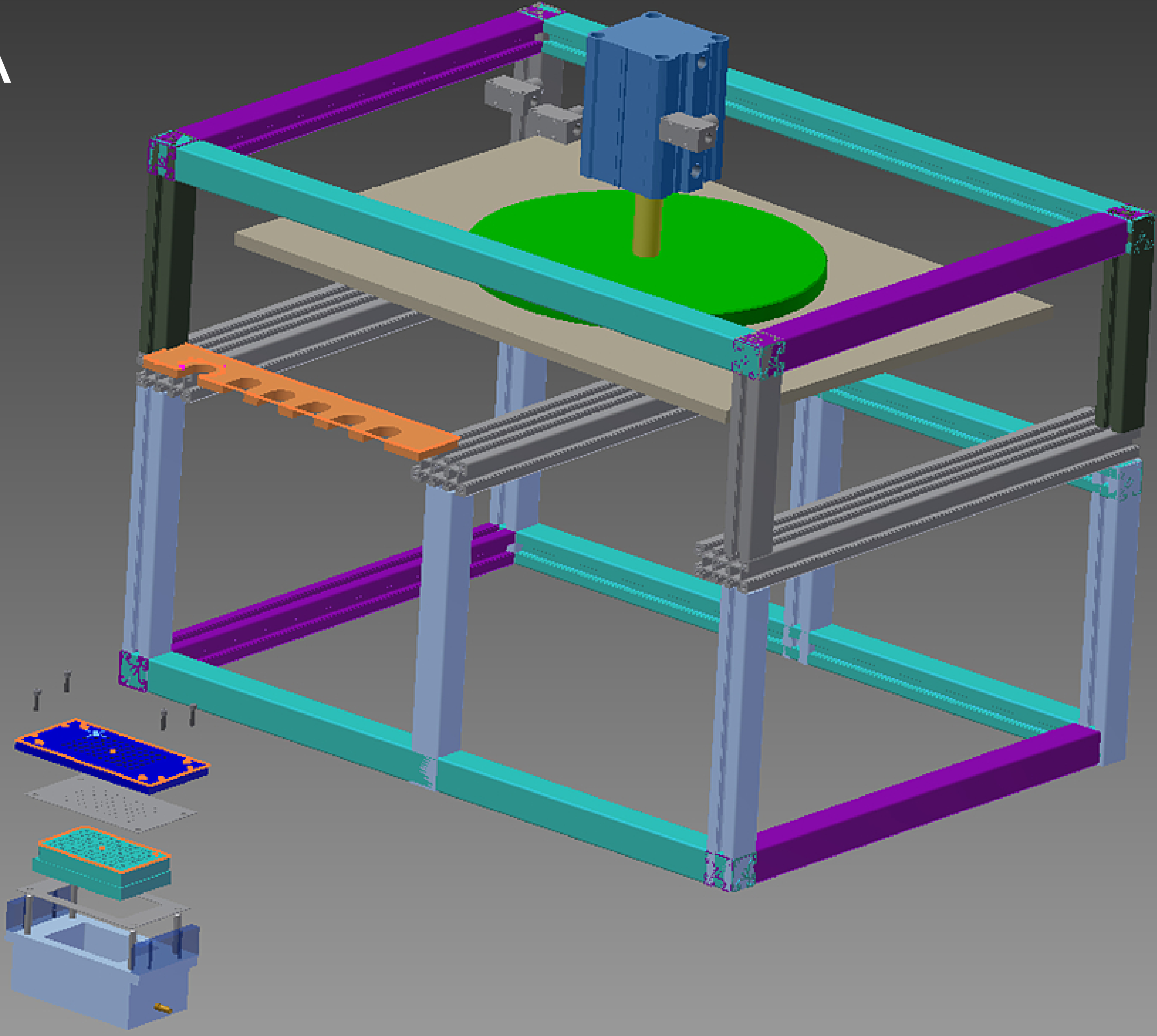

Fig S1 A

Fig S1 B

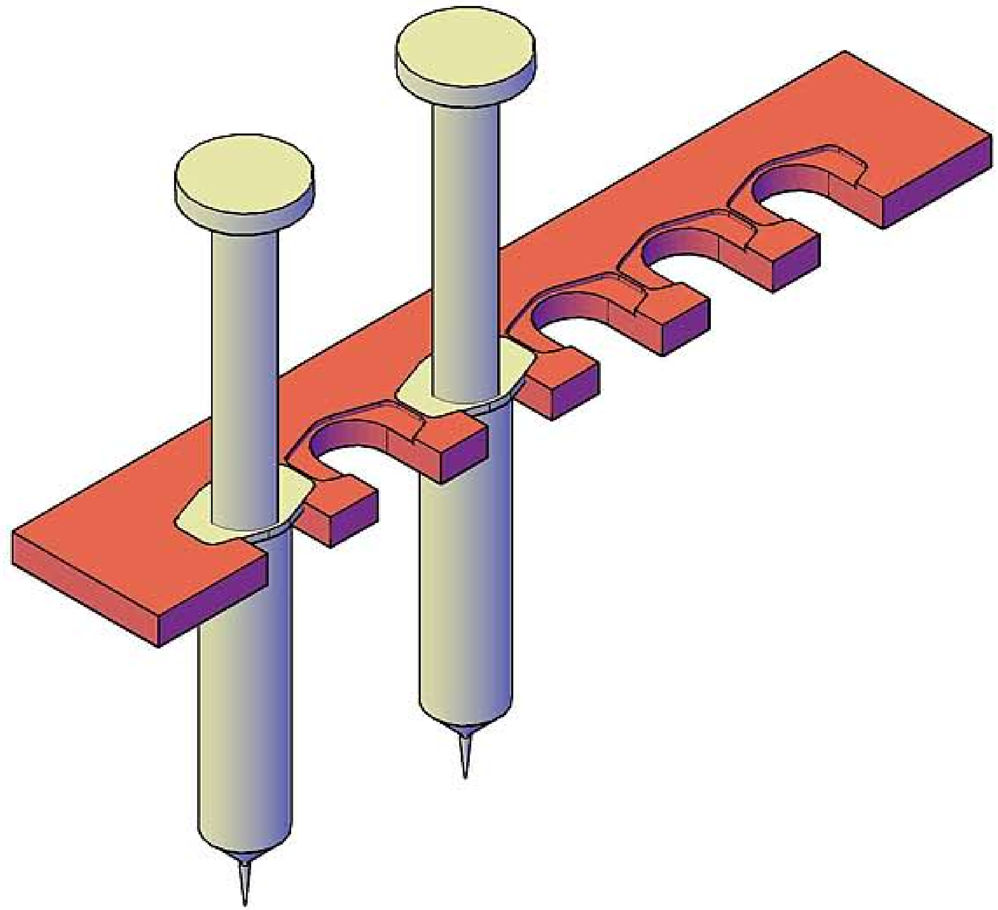

The Wellcome Trust Sanger Institute<br>Genome Campus<br>Hinxton<br>Cambs,BC6 1SA<br>Tel No 01223 834244<br>Fax No 01223494919	Scale NTS<br>Drawn Date 16/06/09<br>Chkd Date CB<br>Appd Date	Tolerance unless othrewise stated<br>Angular +/– 0.25 deg<br>X.XX +/–0.05<br>X.X +/–0.1<br>X +/– 0.5	MATERIAL AL/AL<br>FINISH Clean	PROJECT No WS–10–001	TITLE SYRINGE HOLDER X 6<br>DRAWING No GW –1–Assy<br>REV No A

Fig S1 C

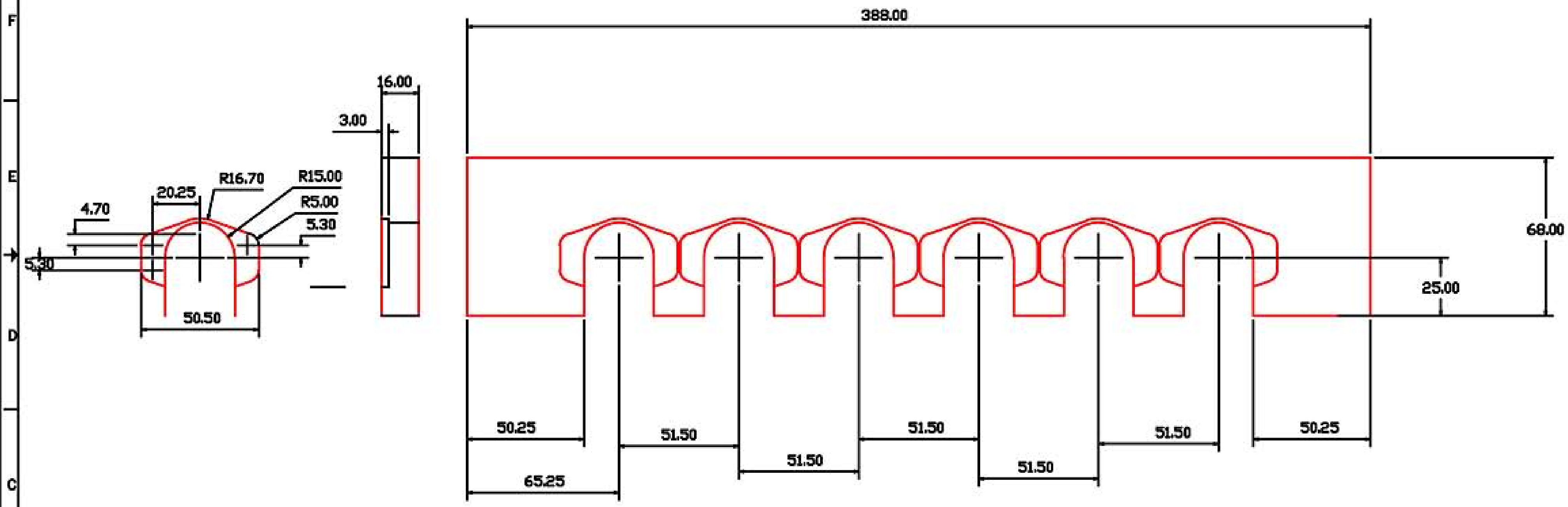

The Wellcome Trust Sanger Institute<br>Genome Campus<br>Hinxton<br>Cambs,BC6 1SA<br>Tel No 01223 834244<br>Fax No 01223494919	Scale NTS<br>Drawn Date 18/06/09<br>Chkd Date CB<br>Appd Date	Tolerance unless othrewise stated<br>Angular +/- 0.25 deg<br>X.XX +/-0.05<br>X.X +/-0.1<br>X +/- 0.5	MATERIAL<br>Tooling Plate<br>FINISH<br>Clean	PROJECT No<br>WS-2010-01	TITLE<br>Syringe Holder<br>DRAWING No<br>2010-01<br>REV No<br>A

Fig S1 D

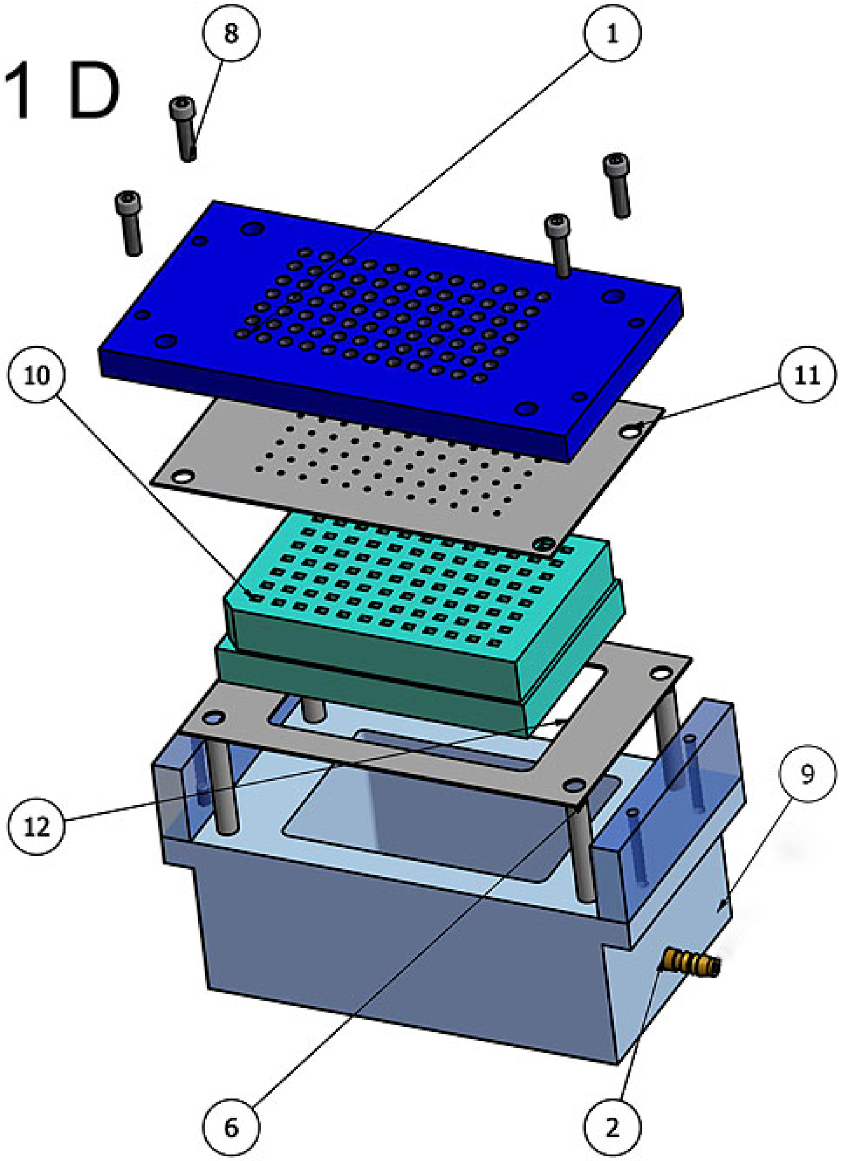

PARTS LIST			
ITEM	QTY	PART NUMBER	DESCRIPTION
1	1	Top plate	
2	1	Barb fitting	
3	2	Side spacers	
4	1	Location Dowels 60lg	
5	1	Location Dowels 60lg_CPY	
6	1	Location Dowels 60lg_CPY1	
7	1	Location Dowels 60lg_CPY_CPY	
8	6	ANSI B18.3.1M - M5x0.8 x 20	Forged Socket Head Cap Screw - Metric
9	1	Lower plate	
10	1	Target Filter Plate	
11	1	Top seal	
12	1	Bottom seal	

Designed by	Checked by	Approved by	Date	Date	
cb4				16/08/2011	

Edition

Sheet 1 / 1

Fig S1 E

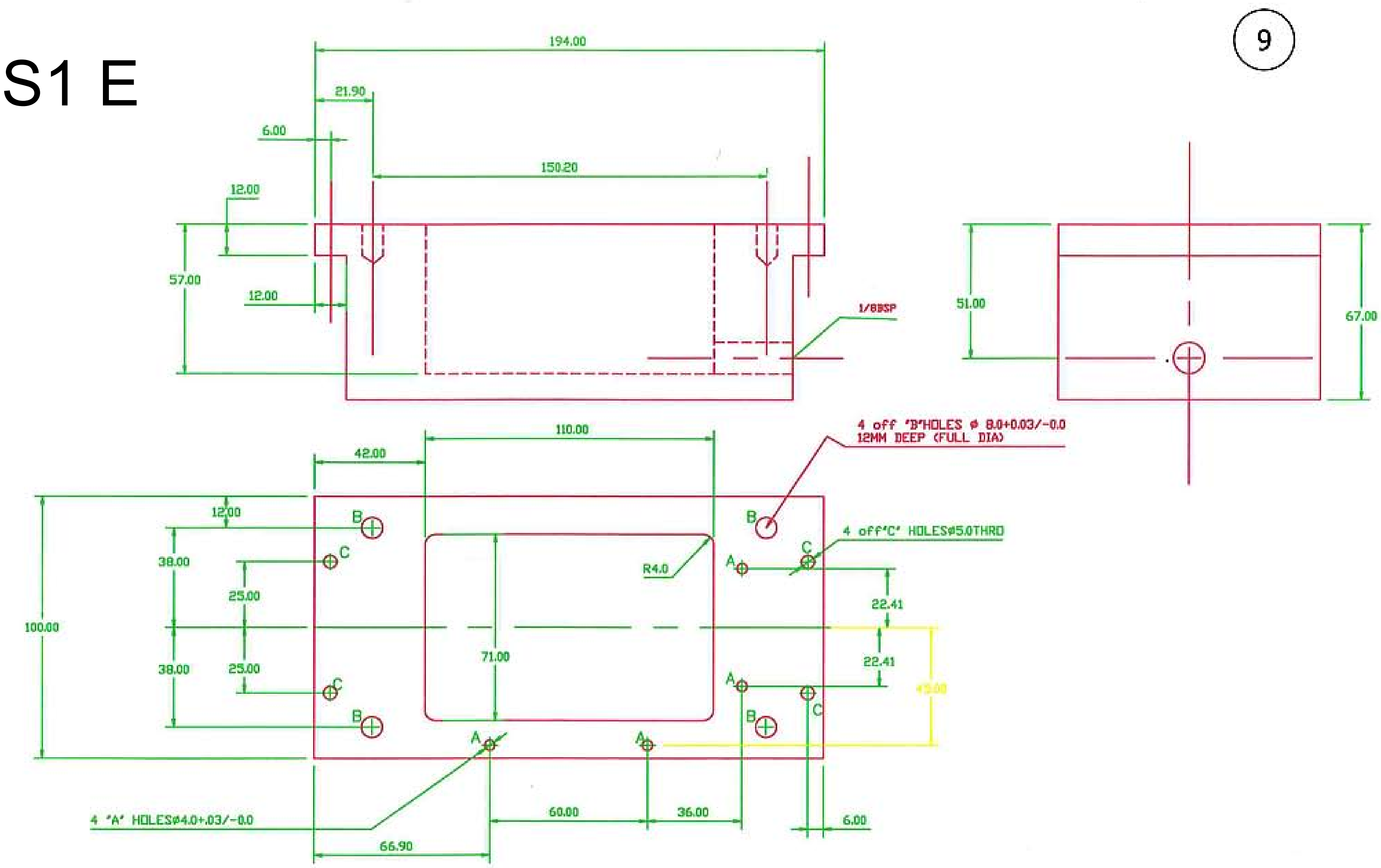

The Sanger Institute<br>Genome Campus<br>Hinxton<br>Cambs,BC6 1SA<br>Tel No 01223 834244<br>Fax No 01223494919	Scale NTS<br>Drawn Date 14/08/08<br>Chkd Date 14/08/08<br>Appd Date 14/08/08	Tolerance unless othrewise stated<br>Angular +/− 0.25 deg<br>X.XX +/−0.05<br>X.X +/−0.1<br>X +/− 0.5	MATERIAL<br>Aluminium Aloy 6082<br>FINISH<br>Anodise	PROJECT No<br>WS−08001	TITLE<br>Lower vac plate<br>DRAWING No<br>0002<br>REV No<br>A

Fig S1 F

12

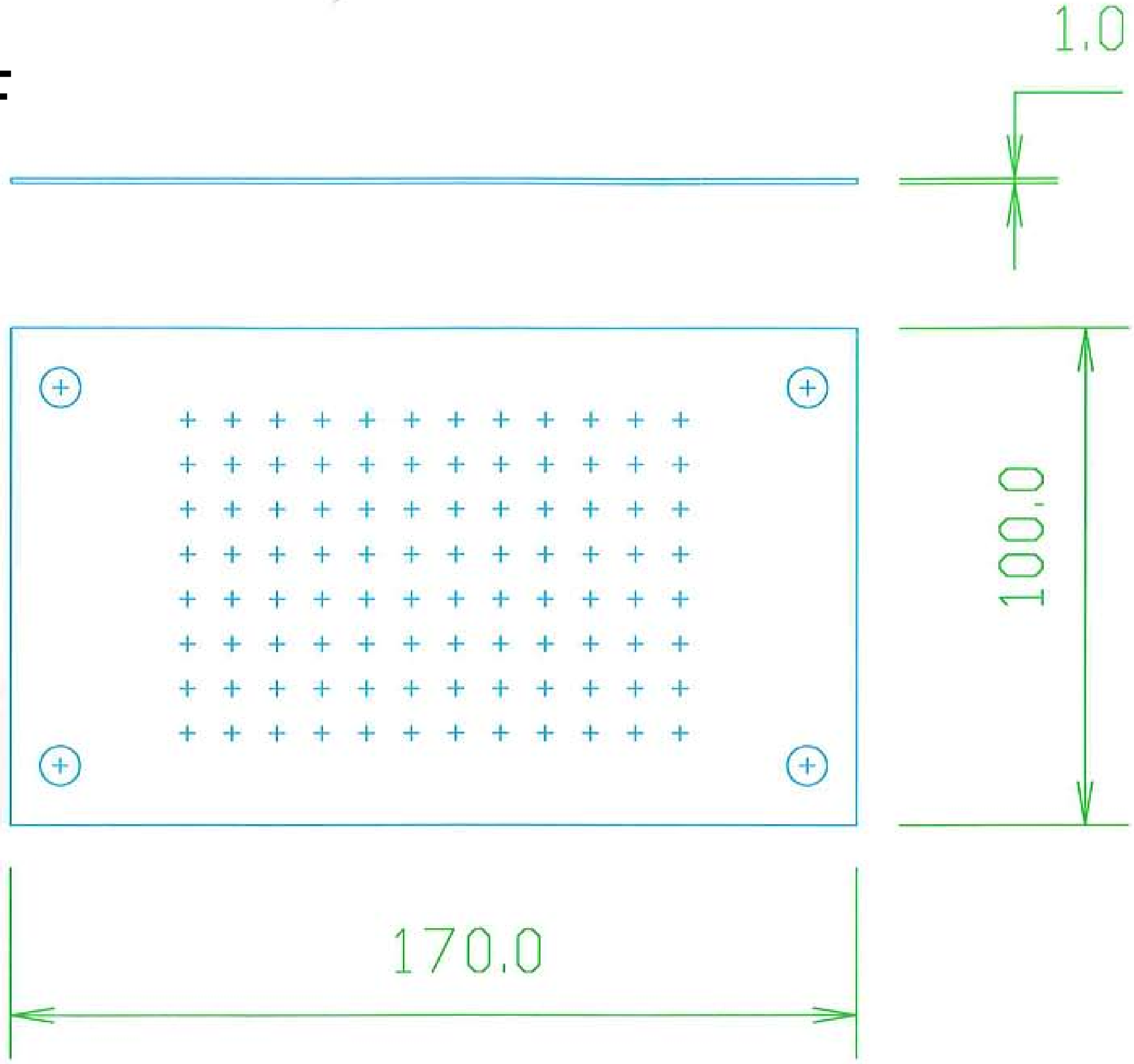

The Sanger Institute<br>Genome Campus<br>Hinxton<br>Cambs,BC6 1SA<br>Tel No 01223 834244<br>Fax No 01223494919	Scale NTS<br>Drawn Date 14/08/08<br>Chkd Date 14/08/08<br>Appd Date 14/08/08	Tolerance unless othrewise stated<br>Angular +/− 0.25 deg<br>X.XX +/−0.05<br>X.X +/−0.1<br>X +/− 0.5	MATERIAL<br>SILICONE SEAL<br>FINISH<br>CLEAN	PROJECT No<br>WS−08001	TITLE<br>TOP GASKET<br>DRAWING No<br>GASKET 1
					REV No

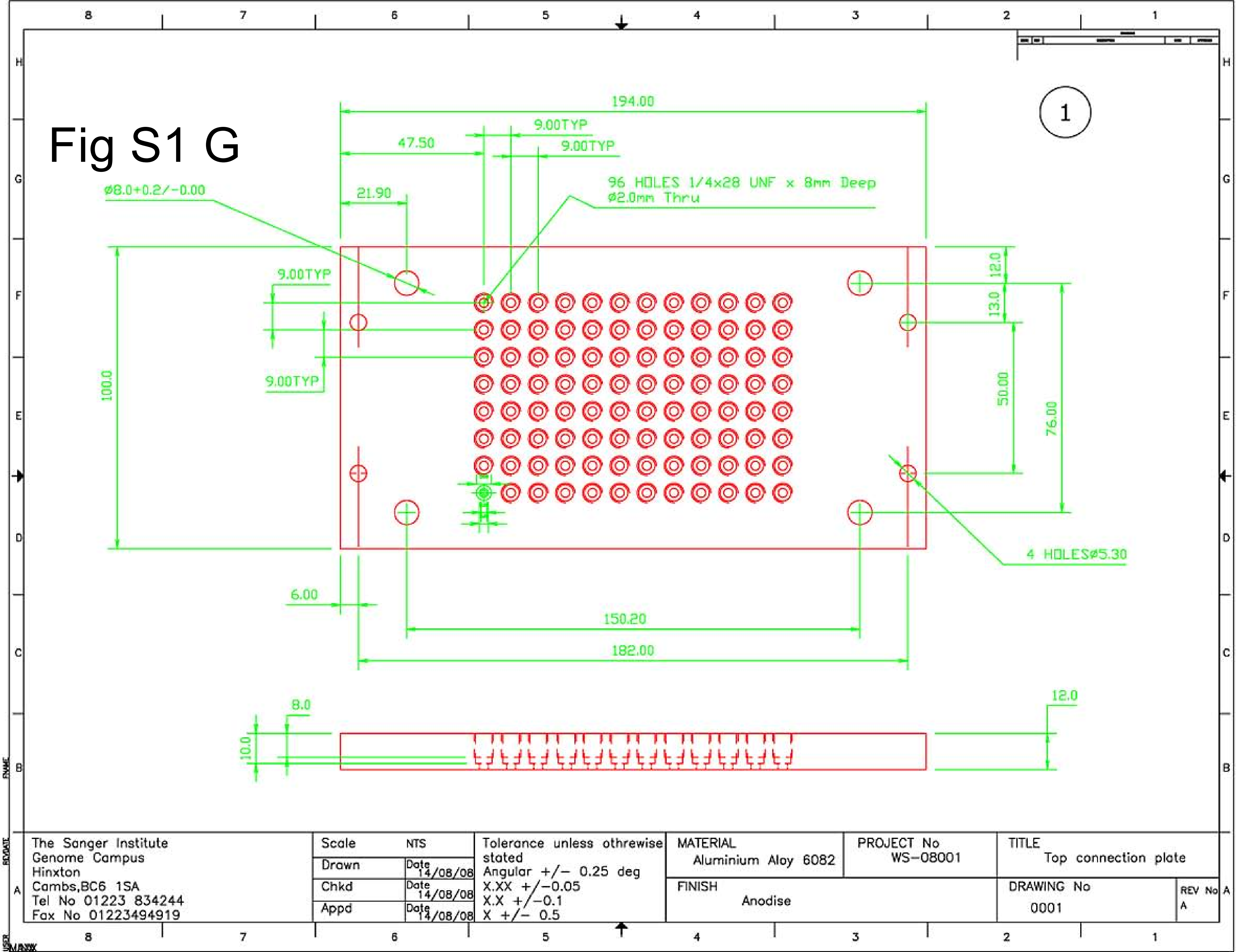
194.00
9.00TYP
47.50
9.00TYP
21.90
ø8.0+0.2/-0.00
96 HOLES 1/4x28 UNF x 8mm Deep
ø2.0mm Thru
9.00TYP
9.00TYP
100.0
12.0
13.0
50.00
76.00
4 HOLESø5.30
6.00
150.20
182.00
8.0
10.0
12.0
The Sanger Institute
Genome Campus
Hinxton
Cambs,BC6 1SA
Tel No 01223 834244
Fax No 01223494919
Scale NTS
Drawn Date 14/08/08
Chkd Date 14/08/08
Appd Date 14/08/08
Tolerance unless othrewise stated
Angular +/- 0.25 deg
X.XX +/-0.05
X.X +/-0.1
X +/- 0.5
MATERIAL
Aluminium Aloy 6082
FINISH
Anodise
PROJECT No
WS-08001
TITLE
Top connection plate
DRAWING No
0001
REV No
A
Fig S1 G

Fig S1 H

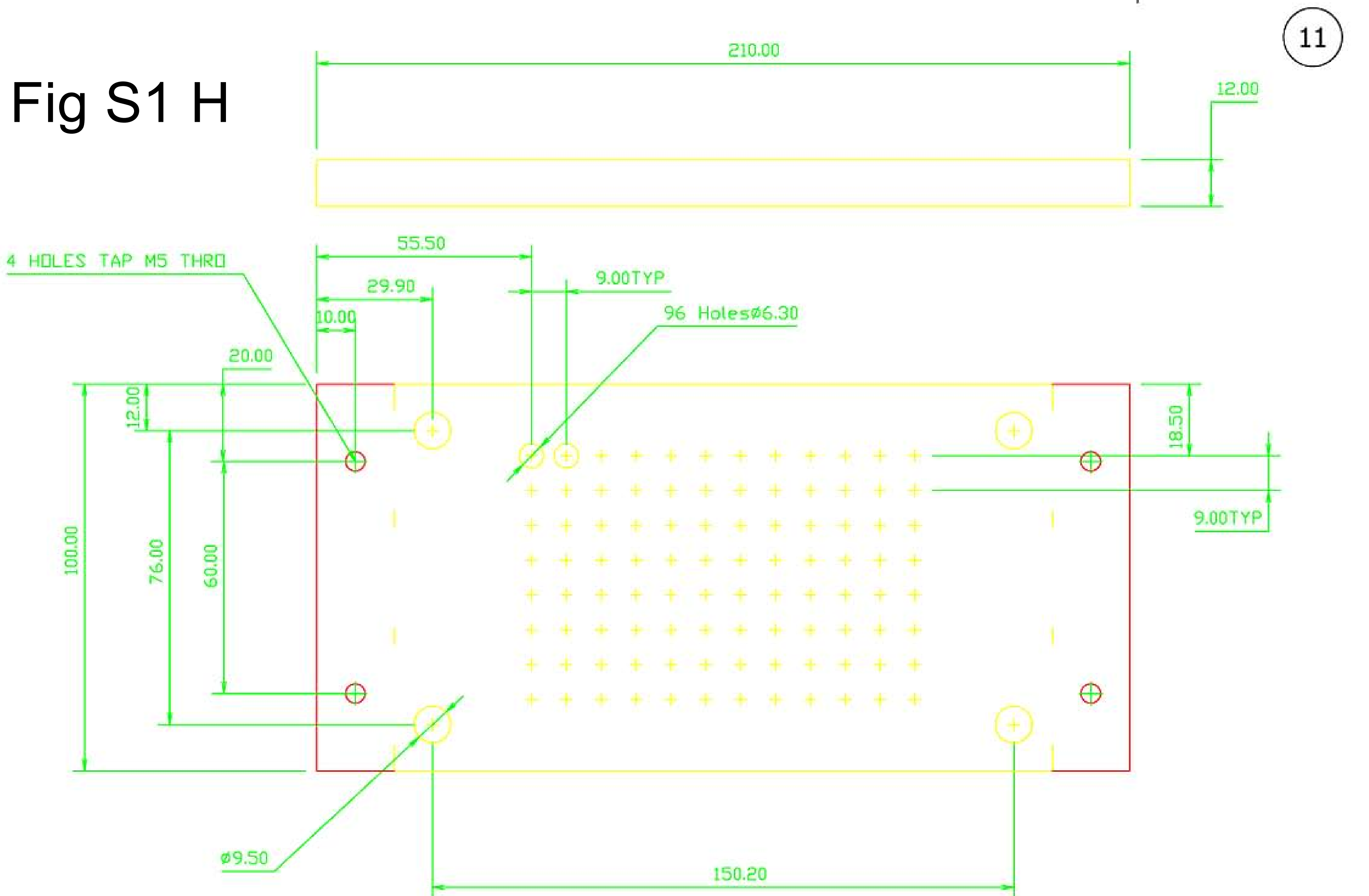

The Sanger Institute<br>Genome Campus<br>Hinxton<br>Cambs,BC6 1SA<br>Tel No 01223 834244<br>Fax No 01223494919	Scale NTS<br>Drawn Date 14/08/08<br>Chkd Date 14/08/08<br>Appd Date 14/08/08	Tolerance unless othrewise stated<br>Angular +/− 0.25 deg<br>X.XX +/−0.05<br>X.X +/−0.1<br>X +/− 0.5	MATERIAL<br>ALUMINIUM ALLOY 6082<br>FINISH<br>CLEAN	PROJECT No<br>WS−08001	TITLE<br>TOP TEMPLATE<br>DRAWING No<br>0004<br>REV No

Fig S1 I

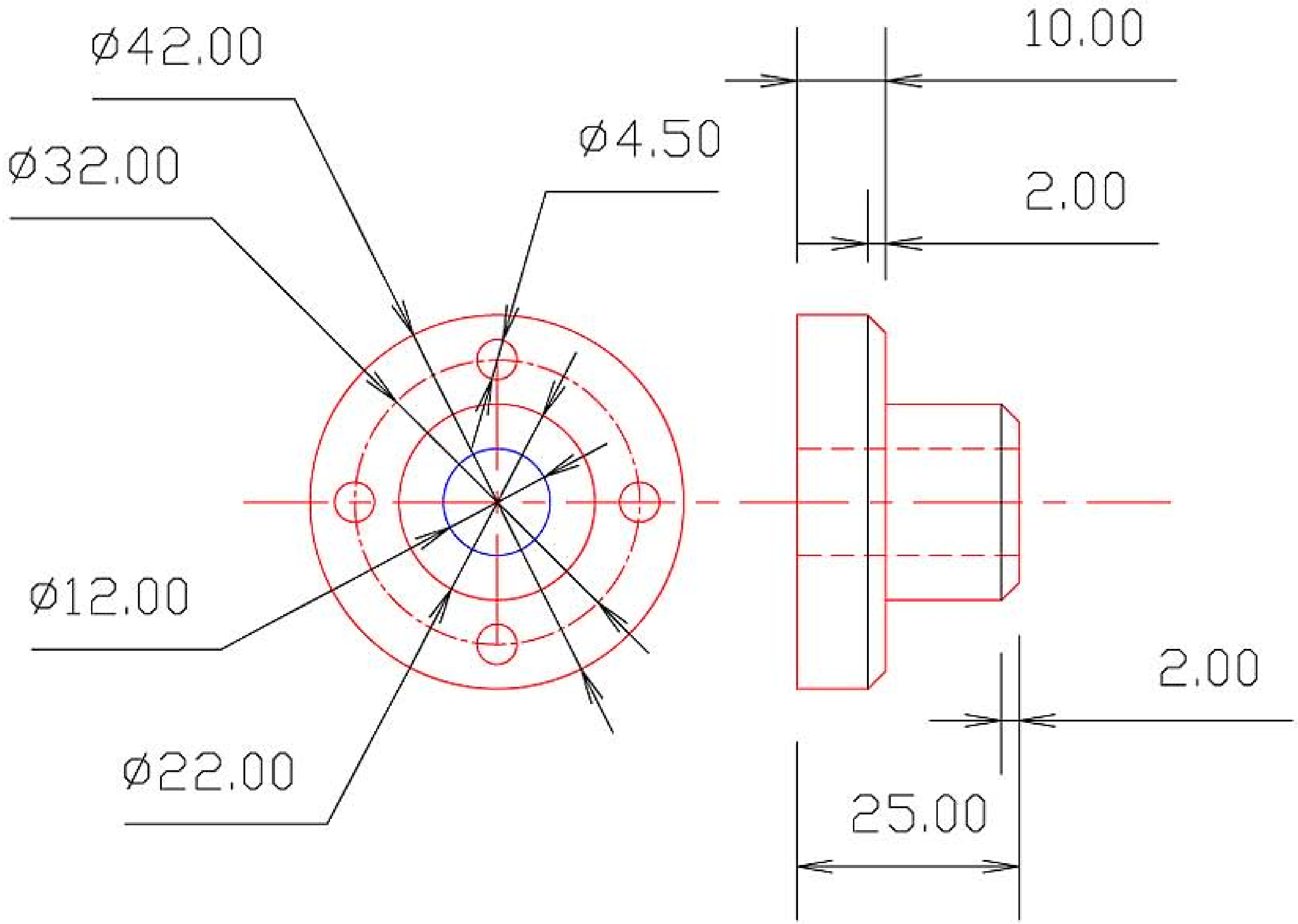

The Sanger Institute<br>Genome Campus<br>Hinxton<br>Cambs,BC6 1SA<br>Tel No 01223 834244<br>Fax No 01223494919	Scale NTS<br>Drawn Date 01/12/08<br>Chkd Date 01/12/08<br>Appd Date 01/12/08	Tolerance unless othrewise stated<br>Angular +/- 0.25 deg<br>X.XX +/-0.05<br>X.X +/-0.1<br>X +/- 0.5	MATERIAL AL/AL<br>FINISH CLEAN	PROJECT No WS-2010-01	TITLE Guide Rod Holders<br>DRAWING No 10-004<br>REV No A
